# Future of digital health and community care: Exploring intended positive impacts and unintended negative consequences of COVID-19

**DOI:** 10.1177/08404704221107362

**Published:** 2022-07-01

**Authors:** Mei Lan Fang, Morven Walker, Karen Lok Yi Wong, Judith Sixsmith, Leslie Remund, Andrew Sixsmith

**Affiliations:** 1 3042 University of Dundee, Dundee, Scotland, United Kingdom.; 2 1763 Simon Fraser University, Burnaby, British Columbia, Canada.; 3 1016 NHS Scotland, Glasgow, Scotland, United Kingdom.; 4 8166 University of British Columbia, Vancouver, British Columbia, Canada.; 5 411 Seniors Centre Society, Vancouver, British Columbia, Canada.

## Abstract

Response to COVID-19 has both intentionally and unintentionally progressed the digitization of health and community care, which can be viewed as a human rights issue considering that access to health and community care is a human right. In this article, we reviewed two cases of digitization of health and community care during the pandemic; one in Scotland, United Kingdom and another in British Columbia, Canada. An integrated analysis revealed that digitization of health and community care has intended positive and unintended negative consequences. Based on the analysis, we suggest five areas of improvement for equity in care: building on the momentum of technology advantages; education and digital literacy; information management and security; development of policy and regulatory frameworks; and the future of digital health and community care. This article sheds light on how health practitioners and leaders can work to enhance equity in care experiences amid the changing digital landscape.

## Introduction

The global technology landscape has changed quickly and persistently. Expedited by the COVID-19 pandemic and fueled by global lockdowns, the pace and scale for the rapid adoption of digital health and community care was dramatic.^1-3^ The COVID-19 pandemic has been a disruptive force that impacted how individuals accessed services and how service providers and systems communicated with each other.^4^

Western nations operating under similar universal healthcare access and delivery systems such as the United Kingdom and Canada have swiftly employed digital healthcare as a way to meet community needs by adapting to changing health (ie, via more eHealth opportunities) and social environments and in relation to the recent restrictions imposed due to COVID-19 (ie, social distancing and restrictions on face-to-face interactions).^5-7^ These changes have allowed healthcare organizations to continue to serve their community through remote working and maintaining essential communication pathways—provider-to-provider and provider-to-user.^8,9^

Prior to the pandemic, transformation of health and community services into a new digital age had progressed steadily, but were generally slow to change.^10^ An unintended positive consequence of COVID-19 was a fast-forwarding of digital health and community care^5^. Given that access to technology is viewed more and more as a human right,^11^ there is a need to ensure that the implications for delivering care digitally are embedded properly, equitably and thoughtfully as part of the new digital norm.

While digital health and community care has the potential to increase the health, autonomy, and well-being of the community through enhancing health information access, maintaining health supports, and enabling social connectedness,^12,13^ significant challenges and barriers still remain, shaped by the need to improve digital literacy and equitable adoption and access.^14^ Consequently, exclusion from the benefits of digital solutions can widen social and health disparities and thus technology at large can be viewed as a social determinant of health.^15,16^ This research-informed discussion piece brings together findings from two studies, an evidence review based in Scotland, the other a service evaluation in British Columbia, Canada. These are presented as case examples aimed at shedding light on the unintended positive and negative impacts of digital healthcare as a by-product of COVID-19.

## A case in the UK: Intended positive impact of digital healthcare in Scotland

In 2021, an evidence review study was conducted to better understand how COVID-19 has impacted existing approaches to healthcare delivery.^5^ Guided by this aim, a scoping review approach was applied, informed by Arksey and O’Malley’s framework six-step protocol.^17^ Two key themes emerged from the analysis: “benefits of digital approaches and technology in healthcare” and “challenges to digital approaches and technology in healthcare.”

Findings highlighted that in the case of Scotland, communication in healthcare had rapidly shifted from face-to-face consultations and meetings to videoconferencing and telehealth following the onset of the pandemic.^5^ For example, in early 2020, before the threat of coronavirus was fully recognized and prior to social distancing and lockdown restrictions, only ∼300 people were using the Near Me videoconferencing App (an NHS funded health consulting service that enables virtual health and social care appointments from any convenient location), as a digital tool for providing health and social care services.^18^ By mid-2020, this figure had increased to 20,000 video appointments via the Near Me App.^18,19^ A year later in July 2021, over 1 million consultations had been conducted through videoconferencing on Near Me.^18,19^ Inevitably, the COVID-19 pandemic acted as a catalyst to accelerate the acceptance of virtual care presumably through the pressure of enforced lockdowns and the fear of contracting the virus in public spaces.

Driven by the health services system, as opposed to the health service users, the impact from the use of virtual consultations helped to maintain healthcare continuity without putting patients and clinicians at risk of COVID-19 transmission.^5^ Another impact from this form of digital healthcare was through the enablement of both health service users and providers to remain at home which removed travel barriers for individuals in challenging health and social situations (ie, older adults with mobility challenges; low-income single parents).^5^ As well, some service users who were previously considered as hard to reach or were socially isolated were now able to access healthcare, while minimizing the cost of travel and limiting the added stress and inconvenience of organizing transport.^5^

It is important to note, however, that older people with fewer digital skills and those with communication problems, such as people with dementia, were less well serviced by the turn to digital health services. Despite this limitation, for clinicians not only in Scotland but worldwide, videoconferencing became the norm for knowledge sharing, and cross-sectoral engagement.^20-23^ The digitized mode of communication facilitated access to the breadth of information globally, transcending boundaries of space, place, and even time thus helping to enhance quality of patient care and thereby enabling better health outcomes for some excluded groups.

Another highly utilized digital platform was NHS Inform.^24^ Individuals, whether requiring immediate healthcare services or not, sought advice and accessed health information via NHS Inform.^24^ The virtual engagement with this platform had increased exponentially since 2018. In April 2018, visits to NHS Inform were 2 million visits; yet by comparison in April 2021, reported on-line visits were 8 million.^19^ Particularly in regards to the pandemic, NHS Inform offered a multitude of services that included evidence-based health information about COVID-19 symptoms and protection, booking PCR tests and the ability to access test results safely and securely.^19,24^ As part of NHS Inform, positive impact was seen in how specific services such as the NHS Helpline 111 enabled individuals to quickly access COVID-19-related medical help, 7 days a week, 24 hours a day.^19,24^ Digital servicing from 111 removed strain and reduced pressure of emergency 999 call handlers.^19^

Nevertheless, there are undoubtedly challenges with the immediate normalization of digital approaches that has quickly shaped: policy; culture of care (clinicians and public); equity in care (digital exclusion); access to care (environmental such as rural digital inaccessibility); health information governance and systems (outdated technology and data protection); and resourcing (lack of equipment and private room space for digital consultations).^25^ The following community-based participatory research study conducted in BC, Canada, highlights some of the unintended negative impacts of digital healthcare that require consideration and scrutiny.

## A case in Canada: Unintended negative consequences of digital community care in BC

In 2020, the 411 Seniors Centre Society and the Science and Ageing Research Institute at Simon Fraser University undertook a community-based participatory^26^ service enhancement study as a response to increasing challenges faced by older community members to engage with community services and supports that had quickly transferred to on-line-only access.^7^ The study was conducted between April and August 2020 in BC, Canada, using a community-engaged, multimethod approach to better understand the digital determinants of health alongside community service recommendations in each of the categories: information and referral services; technology; diverse cultures and languages; poverty, limited resources, and lack of funding; complex system navigation; and volunteers as service providers.^7^

Inescapably, services and supports propelled by the pandemic have rapidly shifted toward digital access.^6^ Findings have shown how the need to engage with digital tools and technologies was no longer a choice but rather a key requirement in order to acquire health information, services, resources, and supports.^7^ Older adults in particular were already experiencing the difficulties of the growing digitization of services, but with the onset of COVID-19, this burgeoning crisis was exacerbated.^7^ For example, some older adults living in BC did not fully benefit from virtual care because they lacked at least one or all of the following infrastructural and social support requirements necessary for access including: wifi, devices and equipment as well as technology knowledge, skills, and self-efficacy. Findings from the study indicated that older adults required all of the above in order to fully benefit from digital healthcare.

The findings had also eluded to how technology challenges of older adults were complex and experienced at multiple levels and, thus, could not simply be resolved by attending computer classes or providing individuals with a device.^7^ Individual-level and community-level barriers identified^7^ were as follows: physical access to technology shaped by limited financial means; limitations of living in remote or rural locations; physical and cognitive impairments that impact ability to use technology; devices and platforms not conducive to age-related challenges; and notably how the pandemic has further placed restrictions for individuals with underlying health conditions still to go outside thereby binding them to digital engagement. At the sectoral level,^7^ fragmented interactions between different sectors with unique goals and mandates were found to be frequently challenged by infrequent and non-transparent communication and lack of integrated servicing which subsequently resulted in initiatives and efforts being misaligned.

## Discussion: A cross-case analysis

An integrated analysis of findings from the two case examples revealed that the impact of COVID-19 on digital health and community care access and use are not clear-cut. The cross-case analysis revealed some similarities and differences between Scotland in the UK and BC in Canada. “Healthcare” itself is broadly similar in multiple jurisdictions. For example, HealthLink BC has offered similar services to NHS Inform during the COVID-19 pandemic. However, the way services are delivered and organized may differ substantially in terms of funding, access, and management of services among other contextual differences. For instance, in terms of healthcare jurisdiction, BC alone has five health authorities, whereas, in the UK, there are 321 NHS trust boards which make up the NHS organisation. The pandemic has been a difficult situation for health providers worldwide, but care provision remains mainly driven by the agendas of service providers and the path dependencies of care systems. These broader structural differences may account for different experiences during the pandemic. They are also important factors in understanding the systems-level and policy-level challenges for the adoption of technology-based products and services in a post-pandemic world.

In particular, systemic and policy barriers were identified in both cases given that the two main forms of virtual care are evident in both Scotland in the United Kingdom and British Columbia in Canada: the first being virtual healthcare service delivery (eg, assessment, consultation, and education),^27,28^ and the second type concerns provision of health-related information via virtual platforms (eg, websites and social media).^29^ The case studies in both the United Kingdom and Canada, supported by a growing literature, suggest similar potential systematic and political barriers that health leadership and management need to pay attention to effect positive digital change and innovation in health service provision.

First, some programs and services do not fully involve key stakeholders (such as those already marginalized in health services) in the design and delivery of services. This can lead to gaps in service implementation and can leave more marginalized populations with poor or no service available to them. Involving the stakeholders is crucial as they will deliver or use the services.^29,30^ However, genuine involvement in the technology development process and technology update is often both challenged by a lack of, or only short-term availability of both human and financial resources. For instance, delivery of digital health and care services need to be supported by appropriate infrastructures, such as available and accessible broadband internet access,^27^ and technology training for service providers and users.^28^

Subsequently, development of evidence on the efficiency and effectiveness of digital healthcare can help to support claims for better, more long-term, resourcing of services, primarily because the success and limitations of some programs are not known. Understanding the nuances of what works, what does not work, why, and in which contexts is crucial for the sustainable development of these digital healthcare programs. A rigorous evaluation of the virtual care programs to include notions of negative unintended consequences and the moral and ethical challenges such programs present is necessary and important.^29,30^

Finally, in Scotland in the United Kingdom and British Columbia in Canada, the roles of the government and stakeholders are unclear in the development of health and care services and in the overall development of virtual care. This can cause confusion in service implementation and delivery. To ensure clarity and inclusion, the roles of the government and the management of services should be clearly defined.^27,29-31^

As Sheikh and colleagues have argued, particularly concerning UK health information technology, national learning across health trusts and UK countries is imperative to better support the equitable and effective development of policy and planning for improved public health and person-centred care.^32^ The disruption brought by COVID-19 and rapid institution of virtual services now requires a re-evaluation of the value, barriers, and facilitating factors of digital health and care strategies and leadership. The case studies presented indicate the need to balance between bottom-up and top-down health technology implementation, and, as Sheikh and colleagues have indicated, improved, “usability and interoperability, developing capacity for handling, processing, and analyzing data, addressing privacy and security concerns, and encouraging digital inclusivity.”

Alongside such priorities, there are also a number of moral and ethical concerns around the prevention of negative unintended consequences of digital health and care,^33^ in the United Kingdom and Canada. Although the benefits of digital services are apparent, there is much improvement to be made to ensure digital equity, particularly among those situated in more disadvantaged and marginalized social positions. To enable more equitable and accessible digital health service development, five key areas are discussed: Building on the momentum of technology advantages, education and digital literacy, information management and security, development of policy and regulatory frameworks, and the future of digital health and community care.

### Building on the momentum of technology advantages

During the pandemic, the use of on-line platforms was essential for the provision of safe and socially distanced care especially for individuals with comorbidities and underlying health conditions.^34^ This mode of engagement was adopted particularly for providing care to patients with a chronic or debilitating illness to enable self-care and manage and monitor their illness and healthcare needs.^35^ Particularly for individuals who may be homebound or living in rural or remote places, the convenience and ease of access to information were found to be the two most important reasons why digital technology continues to be an advantage for retrieving information and resource support. In many instances, digital healthcare has provided more timely connection to necessary and essential services in both the United Kingdom and Canada and across global communities at large. For those who are more socially isolated, or have barriers that impede in-person assistance, on-line engagement can help to ensure that these individuals can reach the supports they need and continue to participate in social activities.

### Education and digital literacy

Barriers to fully digital health and community care are associated with having the appropriate digital training for both service providers and service users. The pace and scale of change brought on by the pandemic was dramatic and healthcare systems had to quickly adapt to changing circumstances.^10^ Digital healthcare, for instance, was implemented to replace the in-person system through video-based appointments and smart technology to limit user-provider interaction—albeit an unintended consequence was that some groups were less able to access digital platforms and technology than others. For example, research has highlighted that older adults were among the least likely group to adapt and use telemedicine and digital healthcare.^36^

The absence and lack of computer skills and lack of self-efficacy when using video-based applications affected peoples’ ability to both arrange and attend appointments or meetings.^37^ According to a recent evidence review,^5^ self-efficacy and confidence when engaging with technology and the components of technological uptake were critical yet limited and unexplored. Accordingly, pre-pandemic, service users and providers who were more reluctant to learn digital skills struggled in the new virtual service world.^37^ Abandonment and non-adoption of digital systems is common with individuals who lack confidence and basic digital skills.^15^ Digital training and education designed to enhance digital skills of people with diverse abilities and needs *on-access* in real time should be a key part of establishing a sustainable system for digital health and community care. Ensuring ease of use accompanied by easy-read instructions is crucial to its adoption success and longevity.

### Information management and security

The pandemic has transformed many aspects of digital health and community care with digital health innovation being rapidly implemented as new virtual models of care.^6,10^ Robbins and colleagues highlight that protecting the vulnerable and high risk of serious illness was the key rationale behind the need for social distancing and innovative digital solutions in healthcare environments.^38^ In the United Kingdom, the drive to find innovative solutions for enabling access and continuity of care during the pandemic accelerated the use of video-based and telehealth approaches as was demonstrated by the Scottish Government and COSLA (2021) strategy. For example, innovation from the technology health sector has been prolific, with new digital tools and packages by clinical software developers such as Egton Medical Information Systems (EMIS) and suppliers of electronic health records, adapting and evolving to shape a range of systems by modifying code, enabling alert tracking, and enhancing infrastructure for more efficient video-based consultations.^38^ Hastened governance and integration of digital solutions was required for safe delivery of digital systems, and without the pandemic to quicken the process, implementation of digital patient management systems would have been significantly slower.^38^

Yet in both the United Kingdom and Canada, there were considerable concerns surrounding the threat of securing patient data when implementing digital health solutions particularly in the healthcare sector. Security and privacy of data is of paramount importance. The notion of “first do no harm” applies also to the evolving digital health landscape to ensure that contents of patient records and histories are kept private and secure from cyber attacks.^39^ Awareness of the cyber security risks and openness to the public is vital to the maintaining the resilience of digital health and community care.^19^ Public trust in digital solutions will progress as the system develops, adapts, and updates to optimize data protection.

### Development of policy and regulatory frameworks

Current UK policy appears, as Asthana and colleagues point out, have exacerbated the digital divide with knock-on negative consequences for digital health outcomes.^40^ To ensure cross country and cross health service learning, national evaluation of digital health and care services is needed, and the lessons learned need to inform policy and regulatory frameworks to support interoperability across technology designs, as well as the development and management of technologies and health information and service systems. Despite the UK National Health Service Long-Term Plan^41^ emphasizing digital transformation to improve health and care access and communication for better health outcomes, continuation of the digital divide militates against equity of care through digital health systems as seen in both the United Kingdom and Canada.^15,42^ Lack of involvement of key stakeholders, especially those in more marginalized groups, in policy development has, arguably, resulted in digital health development and adoption failures. Mandatory consideration of both positive consequences and likely negative unintended consequences for more marginalized population is necessary to enable the moral and ethical application of technology in health settings and contexts.

### Future of digital health and community care

In both the case of Scotland and BC, the benefits and challenges presented are crucial for understanding how a future for digital healthcare can be secured. The rapid incorporation of digital services into healthcare allowing care and communication to continue for service users and service providers has been recognized^37^; yet unwillingness and resistance to full systematic change remains for the various reasons highlighted. There are clear contradictions between the intended positive impacts and the negative unintended consequences. For example, digital engagement can help to alleviate isolation in some cases; however, simultaneously, this was also identified as a barrier to digital healthcare implementation because some individuals preferred face-to-face consultation as a way to alleviate loneliness. Visiting a healthcare provider was often an excuse to leave the house.^37^ Reportedly, social interaction through a video engagement was insufficient for some extremely isolated people.^37^ Hence, implications of findings suggest that a blended approach to healthcare is needed as a way forward. Options for both face-to-face and digital services as and when appropriate should be standardized. In this way, individuals who are more suited to health supports via digital pathways would be able to reap the benefits of digital health services.

Despite the preference for having on-site health and social care programs and face-to-face engagement in the community in BC, virtual services have proven to be an excellent means of maintaining contact and continuing to provide many services and programs.^7^ Collaboration between different health and community-based services and partners, aided by digital tools and technologies, can help both service providers and users in health and social care to expand and enrich their information sources and their reach.^7^ Recommendations to harness the power of digital tools and technologies can include: (i) encouraging both service users and providers to continue outreach beyond the pandemic particularly to more vulnerable members of the community; (ii) developing pilot technology and digital service evaluation projects to maintain momentum started during forced COVID-19 closures; (iii) assessing how relationships were maintained during the lockdown and build on technologies that helped to bring and people together; (iv) offering and maintaining choice for both service providers and service users for how they prefer to engage; and finally ensuring open communication and resource sharing between sectors and organizations that offer health and social services—partnerships are vital for sustainability.

## Conclusion

The progression of digital healthcare and technologies since the beginning of the pandemic has been unprecedented. Indeed, digital systems and applications are beginning to transform the delivery of healthcare globally to meet challenges of increased ageing populations^43^ and complex crises such as the COVID-19 pandemic.^35^ The integrated analysis of findings from the two studies highlighted the tensions and contradictions of the swift onset of digital healthcare as a by-product of COVID-19.

Inarguably, the pandemic has had profound and disruptive impacts on society and on health and care, but there are some positives to take away, and the emergence of digital healthcare has been one. With the need to engage digitally due to COVID-19 restrictions, digital solutions in healthcare and services in the community would be far less advanced. In the past two years, COVID-19 has fast tracked the use of technology in both health and community care settings, often without full consideration of issues of equity. Digital solutions and technologies are minimizing the number of people attending hospital and general practice, through digital solutions that can help to ensure on self-management and self-care in the comfort of one’s home. Nevertheless, there is a still a way to go. As demonstrated in the case of Scotland in the United Kingdom and in the case of BC, Canada, there remains several multi-level barriers to equitable access of digital health and community care that involve accessing digital technologies, considering cognitive and physical limitations of use, addressing issues of cyber security, improving self-efficacy, ameliorating financial and social difficulties, and ensuring rural and remote access.

While virtual care offers huge potential to extend the reach and quality of healthcare, there are potential downsides, particularly in respect to further marginalizing individuals and groups. It is also important to address the acceptance and sustainability of virtual care in a post-COVID world. The implementation of technology needs to be done with consumers and stakeholders, rather than for them, such as through engaged research and development, community consultation, and technology co-production. It is also crucial to address the many structural barriers that exist for jurisdictions and healthcare providers in advancing virtual care, including governance, funding, and service organization.

Despite notable obstacles, digital technology can advance opportunities to allow people who live and work in the community to capitalize on the interconnectedness that digital powers can bring. However, the unintended negative consequences that threaten to replicate and widen inequities must be at the forefront of digital development when shaping the progress of our evolving digital service, alongside a supportive policy and regulatory landscape.

## ORCID iD

Mei Lan Fang https://orcid.org/0000-0003-1990-7537
